# Understanding Emotional Outbursts: A Cross-Cultural Study in Latin American Children with Autism Spectrum Disorder

**DOI:** 10.3390/brainsci14101010

**Published:** 2024-10-08

**Authors:** Maria Cristina Triguero Veloz Teixeira, Rosane Lowenthal, Alexia Rattazzi, Sebastian Cukier, Daniel Valdez, Ricardo Garcia, Gabriela Garrido Candela, Analia Rosoli Murillo, Francislene Pereira da Silva Leite, Giuliana Pinheiro, Kate Woodcock, Justin Cheuk Yin Chung, Carmel Mevorach, Cecilia Montiel-Nava, Cristiane Silvestre Paula

**Affiliations:** 1 Developmental Sciences Graduate Program, Center for Research on Childhood and Adolescence, Mackenzie Presbyterian University, São Paulo 01302-907, SP, Brazil; psicologiagiulianapinheiro@gmail.com (G.P.); csilvestrep09@gmail.com (C.S.P.); 2 Department of Mental Health, Santa Casa de São Paulo School of Medical Sciences, São Paulo 01224-001, SP, Brazilfrancisleneleite@gmail.com (F.P.d.S.L.); 3 Programa Argentino para Niños, Adolescentes y Adultos con Condiciones del Espectro Autista (PANAACEA), Buenos Aires B1640EMQ, Argentinadaniel.valdez@me.com (D.V.); 4 Hospital Pedro de Elizalde, Buenos Aires C1270AAN, Argentina; 5 Facultad Latinoamericana de Ciencias Sociales (FLACSO), Buenos Aires C1026AAC, Argentina; 6 Facultad de Medicina, Departamento de Psiquiatria y Salud Mental de la Infancia y la Adolescencia, Universidad de Chile, Santiago de Chile 8380453, Chile; ricardo.garcia.sepulveda@gmail.com; 7 Facultad de Medicina, Clínica de Psiquiatría Pediátrica, Universidad de la República, Montevideo 118000, Uruguay; gabrielagarridof86@gmail.com; 8 Organización Estados Iberoamericanos para la Educación, la Ciencia y la Cultura (OEI), Santo Domingo 10108, Dominican Republic; 9 Centre for Applied Psychology, School of Psychology, Institute for Mental Health, University of Birmingham, Birmingham B15 2TT, UK; contact@katewoodcock.com; 10 School of Psychology, University of Birmingham, Birmingham B15 2TT, UK; justincychung@googlemail.com; 11 Centre of Human Brain Health, School of Psychology, University of Birmingham, Birmingham B15 2TT, UK; c.mevorach@bham.ac.uk; 12 Department of Psychological Science, University of Texas Rio Grande Valley, Edinburg, TX 78539, USA

**Keywords:** autism spectrum disorder, emotional dysregulation, emotional outbursts, cultural similarities

## Abstract

Objectives: One of the behavioral problems strongly associated with emotional dysregulation (ED) in ASD is emotional outbursts (EOs) characterized by a pattern of challenging behavior that varies across individuals and across time. Cultural factors can modulate the expression of EOs. This study aimed to characterize the profile of emotional outbursts (EOs) in children with autism spectrum disorder (ASD) across various countries in Latin America and to identify clinical, emotional, and contextual factors that contribute to the onset and frequency of EOs within this diverse sample. Methods: A cross-sectional and cross-cultural study was conducted between 2023 and 2024 comprising samples from five countries in the Latin American Network for Autism-REAL: Argentina, Brazil, Chile, Uruguay, and the Dominican Republic. We studied 689 children with ASD (age x = 8.7 ± 2.6 years) using the Emotional Outburst Questionnaire (EOQ). Results: We identified different types of EO among children with ASD in our sample. The most frequent was the ‘behavioral indicators of emotion’ (52.0%) followed by ‘increased motor activity’ (28.3%), ‘non-speech vocalizations’, (27.6%), ‘mild verbal aggression’ (23.8%), and ‘avoidance’ (21.5%). Children in a bad mood or having a bad day or experiencing irritability were the most significant factors that increased the likelihood of EOs. Conclusions: Our results revealed that irritability is an important trigger for EOs and should not be disregarded or underestimated when monitoring the mental health of children with ASD.

## 1. Introduction

Autism Spectrum Disorder (ASD) is a complex neurodevelopmental disorder defined by a spectral presentation of social communication and social interaction impairments in multiple contexts with a predominance of deficits in socio-emotional reciprocity; deficits in verbal and non-verbal communication skills used for social interaction; and deficits in developing, maintaining, and understanding relationships, as well as restricted and repetitive patterns of behavior, interests, or activities [1].

ASD symptoms cause clinically significant impairments in different areas of social, school, family, and professional functioning in adulthood [2]. In the course and evolution of ASD, scientific evidence reports that emotional dysregulation (ED) is an important sign that must be identified and treated as soon as the first symptoms manifest themselves in the child’s behavioral repertoire [3]. ED is a broader term that describes a chronic difficulty in managing and responding to emotional experiences in a socially acceptable manner and could be considered a core dimension of psychopathology in different neurodevelopmental and psychiatric disorders [4].

There is no agreement on the ED definition. Bunford et al. [5] proposed an operational definition considering it as the inability of an individual to exercise any or all aspects of the modulatory processes involved in emotion regulation, to such a degree that the inability results in the individual functioning meaningfully below his or her baseline. It includes a range of problems related to experiencing, processing, and expressing emotions [6]. According to previous authors, the main alterations of ED are an abnormal and excessive emotional responses in comparison to social norms, uncontrollable and rapid fluctuations in emotions, and an aberrant allocation of attention to emotional stimuli [5,7].

In individuals with ASD, ED is prevalent, persistent, and clinically significant, contributing to functional impairment [8]. The study of ED in this neurodevelopmental disorder has grown in recent years [9]. One of the behavioral problems strongly associated with ED in ASD is emotional outbursts (EOs) [10]. According to Chung et al. [10], EOs are highly emotional, explosive episodes, characterized by a pattern of challenging behavior that varies across individuals and across time, but can be immediately identified by caregivers. The EOs are exaggerated episodes of anger or frustration that are often disproportionate to the context [10,11]. EOs are characterized by abrupt and intense displays of emotion, such as anger, frustration or distress, often involving behaviors like crying, yelling, or verbal and physical aggression [10,12,13]. These outbursts are multidetermined and result from maladaptive responses to various stimuli [14].

The expression of the EO can vary depending on contextual factors and individual differences in how emotions are managed and expressed [15,16]. Previous studies referred to EOs as “temper outbursts” or “tantrums” but other synonymous terms include “meltdowns” and “rages” [10,17]. The manifestation of EOs in individuals with ASD is well-documented, with irritability identified as a prominent symptom linked to these episodes [12,18,19]. Over the past decade, the significance of irritability in ASD has gained increased attention. Irritability in individuals with ASD often occurs within the context of emotional dysregulation, and it includes both mood components (such as being moody or angry) and behavioral components (such as emotional outbursts) [14].

Irritability is characterized by a heightened sensitivity to stimuli that result in frequent feelings of frustration, anger, or annoyance and represents a low threshold for emotional reactions to perceived stressors or challenges [20]. This can be due to internal factors (such as sensory sensitivities or difficulty in processing emotions) or external factors (such as changes in routine or challenging social interactions) [21]. Irritability is considered a form of ED as it involves difficulties in managing negative emotional responses and can significantly impact daily functioning, particularly in autistic individuals. Carlson et al. [22] suggest that irritability and EOs should be distinctly defined and studied separately. They emphasize the importance of understanding the relationship between these constructs to improve targeted interventions. By exploring irritability in individuals with neurodevelopmental conditions, including ASD, researchers can determine whether irritability precedes EOs or if the lack of control during an EO leads to subsequent mood changes. This nuanced approach could significantly enhance our understanding and management possibilities of these symptoms in neurodevelopmental disorders.

Among the contextual factors impacting the expression, frequency, and intensity of the EO, culture is among the most important ones. Cultural norms dictate how emotions are expressed and regulated. In cultures where emotional restraint is valued, children with ASD displaying intense EOs might be viewed more negatively or as demonstrating challenging behavior. In contrast, these behaviors might be interpreted differently [23]. Consequently, the cultural implications for EOs in children with ASD highlight the importance of understanding how different cultural contexts influence perceptions, responses, and support for these behaviors [24]. Cultural factors such as beliefs about disability, norms regarding emotional expression, and access to resources can significantly impact how EOs are perceived and managed in children with ASD [25]. Latino cultures often emphasize strong family ties, respect for authority figures, and emotional expressiveness within familial contexts [26]. These cultural values may influence how EOs in children with ASD are perceived within Latino families and communities. Research suggests that Latino parents of children with ASD may use culturally specific parenting practices, such as familism (prioritizing family unity and support) and obedience (respect for authority and elders), which can influence how EOs are managed and understood [27]. In a population-based study, Becerra et al. [28] found that children with ASD born to U.S. African American/Black, foreign-born Black, foreign-born Central/South American, and U.S.-born Hispanic mothers were at a higher risk of exhibiting severe EOs and impaired expressive language than children of U.S.-born white mothers. 

Chung et al. [12] studied the role of culture in ED, showing some differences in EOs across two samples of caregivers of children and young people (ages 6–25) in Brazil and the UK with ASD, Down’s syndrome, or intellectual disability. According to the Brazilian caregivers’ reports, the most notable difference was in the triggers of EOs’ related to ‘threat to self’, grouped in the ‘perceived safety cluster’. This EO score is related to the perception of one’s safety and helps to identify how environmental or contextual factors contribute to an individual’s feelings of safety or vulnerability. According to the authors, the difference may be related to the motivation for a person to mask their emotions to hide characteristics that others may perceived as less socially desirable, showing how the individuals may also receive more prejudice and stigma from the community that may negatively impact their self-esteem [12].

Despite the importance of culture in understanding the expression, frequency, and correlates of EOs in the ASD population, research in Latin America remains markedly limited, reflecting a significant gap in the region’s scientific inquiry into neurodevelopmental disorders [29,30]. Compared to more developed regions, such as North America and Europe, Latin America faces substantial challenges in funding, infrastructure, and trained specialists dedicated to ASD research [31]. This disparity results in a dearth of culturally relevant studies that could illuminate unique environmental and social factors influencing EOs among Latin American ASD populations. The few studies conducted in Latin America often struggle with small sample sizes, heterogeneous methodologies, and inadequate dissemination, hindering the advancement of evidence-based practices and tailored interventions for individuals with ASD across the region [32]. Efforts to address these disparities are crucial to fostering inclusive global research initiatives that can better serve the diverse needs of individuals with ASD in Latin America.

To comprehensively explore the predictors of the frequency and intensity of EOs in children with ASD residing in Latin America, it is important to consider their unique contextual factors, cultural backgrounds, and societal constraints, which extend beyond the characteristics of the condition itself. Therefore, this study draws data from Argentina, Brazil, Chile, the Dominican Republic, and Uruguay—countries in Latin America characterized by a blend of Spanish and Portuguese colonial legacies intertwined with indigenous and African cultural influences. Shared traditions in music, cuisine, and strong familial bonds underscore the cultural similarities across these nations [33]. However, significant health disparities persist among marginalized groups such as women, children, adolescents, and indigenous populations, according to UNICEF [34], highlighting the broader societal challenges that may impact the emotional regulation of children with ASD in these diverse settings.

An accurate assessment of EOs as a measure of ED must encompass frequency, duration, and intensity, as well as associated contextual factors [35]. However, studies aimed at evaluating EO topographies as well as associated contextual factors are scarce in developing countries when compared to developed countries [36,37,38].

This study aims to characterize the profile of emotional outbursts in children with ASD across various countries in Latin America. It seeks to identify clinical, emotional, and contextual factors contributing to EO onset and frequency within this diverse sample. By encompassing data from multiple Central and South American nations, the research offers a unique perspective on EO experiences among individuals with ASD in the region. This study not only provides empirical insights into EO dynamics, but also advocates for culturally responsive regional strategies in policy and practice, addressing the diverse needs and contextual nuances of individuals on the autism spectrum across Latin America.

## 2. Materials and Methods

This is a cross-sectional and cross-cultural study conducted between 2023 and 2024 comprising samples from five countries of the Latin American Network for Autism-REAL: Argentina, Brazil, Chile, Uruguay, and the Dominican Republic.

### 2.1. Participants

The sample was composed of caregivers of 689 children with ASD (mean of age 8.7 ± 2.6; 37.0% from Argentina, 21.9% from Brazil, 18.9% from Uruguay, 15.2% from Chile, and 7.4% from the Dominican Republic). The inclusion criterion for the children was the diagnosis of one of the following conditions registered in the clinical report informed by the caregivers (autism, ASD, Asperger’s syndrome, Pervasive/pervasive developmental disorder not otherwise specified).

### 2.2. Measures

The Emotional Outburst Questionnaire/EOQ: It is a questionnaire based on the informant-report and contains 133 items distributed into three sections detailed by Chung et al. [10]. Sections 1 and 2 assess types of EO including behavioral composition/topography (i.e., ‘behavioral indicators of emotion’ such as angry or annoyed facial expressions, crying, signs of distress and whining; ‘increased motor activity’ such as flailing arms, non-directed kicking, pacing, repetitive behaviors, rushing about, stamping feet and tics; ‘non-speech vocalizations’ such as making sounds or noises; ‘mild verbal aggression’ such as insults, name-calling, screaming, shouting, and swearing; ‘avoidance’ such as dropping to floor, going to room, leaving situation, running away, among others), frequency, duration, intensity, and recovery duration of outbursts. Section 3 assesses setting events and antecedents related to outbursts, behaviors that occur after outbursts, and caregiver management strategies effective in stopping outbursts [10]. The frequency of the EOs was scored on a three-point scale: Not applicable/Never/Rarely (0–3 times out of 10), Sometimes (4–6 times out of 10), Often/Always (7–10 times out of 10). The duration and recovery duration of EOs were scored on a seven-point scale: Fewer than 5 min, 5–15 min, 15–30 min, 30 min to 1 h, 1–2 h, 2 h to a day, and a day or more. The intensity of EOs was scored in two types: most severe and least severe. The 55 items related to the antecedents and setting events of outbursts were rated on a three-point scale: Not applicable/Never/Rarely (0–3 times out of 10), Sometimes (4–6 times out of 10), Often/Always (7–10 times out of 10). In the English study, most contextual items loaded onto six latent factors, with the internal consistency as indicated by Cronbach’s α ranging from 0.68 to 0.84 [10]. The translation and cross-cultural adaptation of the Brazilian Portuguese EOQ was carried out in a previous study [39]. The predictive validity of the Portuguese version of the EOQ using an external criterion showed excellent indicators of sensitivity to measure changes influenced by the individual’s environment context, which influences any type of intervention, as well as parent’s mental health [39]. For this study, we conducted the translation and cross-cultural adaptation from the English version of the EOQ to Spanish, following the stages recommended by the International Test Commission [40]. This process was composed of three stages: translation and cross-cultural adaptation, back-translation, final modifications, and evaluation by target audience. After translating, back-translation analyses for modifications were performed based on identifying idiomatic, conceptual/experiential, and semantic discrepancies for the synthesis composition. Out of 133 items, 3 (2.25%) needed revision, but only 2 were partially modified, according to idiomatic criteria. After reviewing the items, the agreement index related to the quality of the translation among four mental health professionals and four parents of children with ASD from Argentina and Uruguay (the agreement index between the mental health professionals was 0.71 and 1 for the parents). These indices met the content validity parameters of acceptability [40,41]. The EOQ can be accessed in full in the supplementary information of this publication: https://www.nature.com/articles/s41598-022-11474-4, accessed on 17 September 2024.

### 2.3. Sociodemographic and Clinical Questionnaire

The questionnaire assessed characteristics of the children’s families regarding age, education level, and relationship of the caregiver who responded to the EOQ. The child’s age, sex, education, diagnosis, associated medical conditions, level of verbal ability, level of intellectual disability, and age at which mental health interventions began after diagnosis of ASD were assessed, as well as the types and hours of treatments the child currently receives. This information was adapted from the REAL questionnaire used by Paula et al. [42].

### 2.4. Data Collection Procedure

Caregivers from the five countries provided informed consent prior to participating in this study. The 689 parents and caregivers (from Argentina, Brazil, Chile, the Dominican Republic, and Uruguay) completed the Emotional Outburst Questionnaire and the Sociodemographic and clinical questionnaire online on the Research Electronic Data Capture (REDCap) platform [43]. The data collection was online and for this purpose, five links were generated on the REDCap, one for each country. The study was publicized for the recruitment of participants by each country’s researchers on the institutions’ and researchers’/authors’ social media.

### 2.5. Data Analysis

Initially the data were analyzed descriptively. For categorical variables, absolute and relative frequencies were presented, and for numerical variables, summary measures (mean, standard deviation, quartiles, minimum, and maximum). For the analyses, we verified the distribution of the variables using the Kolmogorov–Smirnov test. The caregiver’s age had a normal distribution and was treated with a parametric test, while the other variables did not have a normal distribution and were treated with non-parametric tests. The associations between the categorical variables were verified using the Chi-Square test or Fisher’s exact test. Comparisons of means between two groups and more than two groups were performed via Student’s *t*-test and Analysis of Variance (ANOVA), respectively. Both the Student’s *t*-test and ANOVA present normality in data distribution as one of the assumptions, which was verified via the Kolmogorov–Smirnov test. In case of violation of this assumption, the Mann–Whitney test (comparison of means of two groups) and Kruskal–Wallis (comparison of means between more than two groups) were alternatively used. When differences in means were verified in the Kruskal–Wallis test, distinct groups of means were identified via Dunn–Bonferroni multiple comparison tests to maintain the global significance level. For the 1-way ANOVA (1 fixed factor (locality/country), Duncan’s multiple comparison tests were used.

An ordered logit regression model with random effects was used to evaluate the effects of family, demographics, and clinical characteristics as well as the child’s EOs’ antecedents and setting events on the frequency of EOs [44]. For the ordered logit regression model, we selected the types of EO that occurred with a frequency equal to or greater than 15% in the point scale ‘Often/Always’, and for the antecedents and setting events (contextual factors) associated with these EOs we selected the antecedents (triggers) that occurred with at least 25% frequency in the ‘Sometimes’ and ‘Often/Always’. The regression model with random effects incorporates the effect of each country in the form of a random effect, accommodating a possible dependence between the observations of young people living in the same location. For this analysis, the effects of the variables sex of the child (adopting the male sex as a reference), the child’s age in years, and the caregiver’s education level (adopting the higher education level as a reference) were controlled. The experimental variables used for the logit regression model were clinical, emotional, and contextual factors. The clinical factors were level of verbal ability, intellectual disability, age at diagnosis of ASD, age at start of treatment and hours per week of treatment. The emotional and contextual factors were bad mood or having a bad day; not being given or not being able to do something the person, being fixated on a thought or idea, tired, having to wait before being given or being able to do something, doing a difficult task, being told off criticised or accused of making a mistake, change in expectation, being asked to do something the person may or may not want to do, being teased, hungry or thirsty, disagreement with others, change in own routine. The ordered logit model corresponds to the analog of logistic regression for ordinal polytomous responses. Furthermore, hierarchical selection was adopted in this study, which allows for structuring the investigation of risk factors and facilitates interpretation. The difficulty variables (verbal ability and intellectual disability) were considered as level 1 predictors of hierarchical selection. Next, as level 2, the diagnosis and treatment variables. For levels 3 and 4, the EO antecedents and setting events and the child’s demographic and family characteristics were considered as predictors, respectively. For all statistical tests, a significance level of 5% was used. The analyses were carried out using the SPSS 20.0 (IBM Corp. Released, 2011, Armonk, NY, USA) and STATA 17 statistical packages [45].

## 3. Results

### 3.1. Sociodemographic and Clinical Variables 

As exhibited in Table 1, most of the participants lived in Argentina (37.0%, n = 255), followed by Brazil (21.9%, n = 151), Uruguay (18.4%, n = 127), Chile (15.2%, n = 105), and the Dominican Republic (7.4%, n = 51). There were significant differences among caregivers in terms of educational levels (*p* < 0.001), relation to the child (*p* = 0.006), and age of the respondent (*p* = 0.003) and the child (*p* < 0.001). Specifically, Chilean (59.8%) and Argentinian (42.5%) parents have higher educational levels compared to other countries. Most participants from all countries self-identified as mothers, and Argentina had the lowest average age of children compared to other countries (average = 8.0 years, SD = 2.0). There were different distributions of children’s diagnosis (*p* < 0.001), other neurological or genetic problems diagnosed (*p* = 0.002), level of verbal ability (*p* < 0.001), intellectual disability (*p* = 0.009), and age of the diagnosis (*p* < 0.001) by country. Brazil had one of the highest percentages of children with the diagnosis of autism or ASD (95.3%) followed by the Dominican Republic (92.1%) compared to other countries. Argentina had the lowest age at diagnosis (average = 3.1 years old, SD = 2.8), and Chile the highest (average = 5.3 years old, SD = 4.1). Related to verbal skills, Brazil had the highest percentage of children whose parents reported using complex sentences (64.8%). In contrast, children from the Dominican Republic had the highest percentage of non-speaking children (26.0%) and using only isolated words to speak, along with Uruguay (just over 20.0%). Regarding reports on intellectual disability, the Dominican Republic had the highest percentage of children reported with indicators of intellectual disability (44.9%) compared to other countries (less than 25.0%). Although similar, the average age at starting treatment in Brazil was lower than in Uruguay, Chile, and the Dominican Republic. There were no differences in this variable between Argentina and other countries.

### 3.2. Emotional Outburst Profile

**Figure 1:**
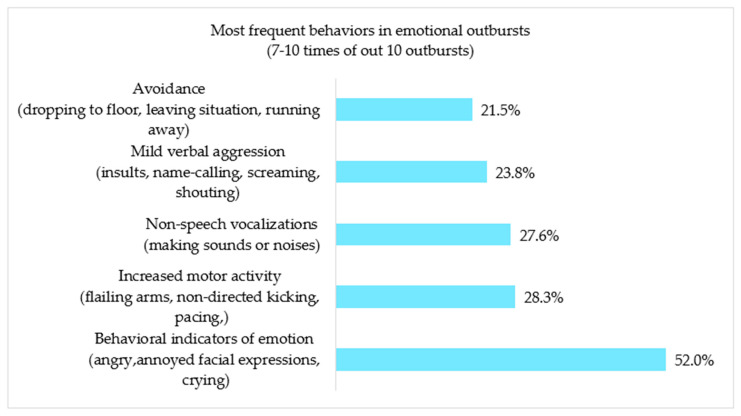
Distribution of the main emotional outbursts in the same sample (n = 689).

**Figure 2:**
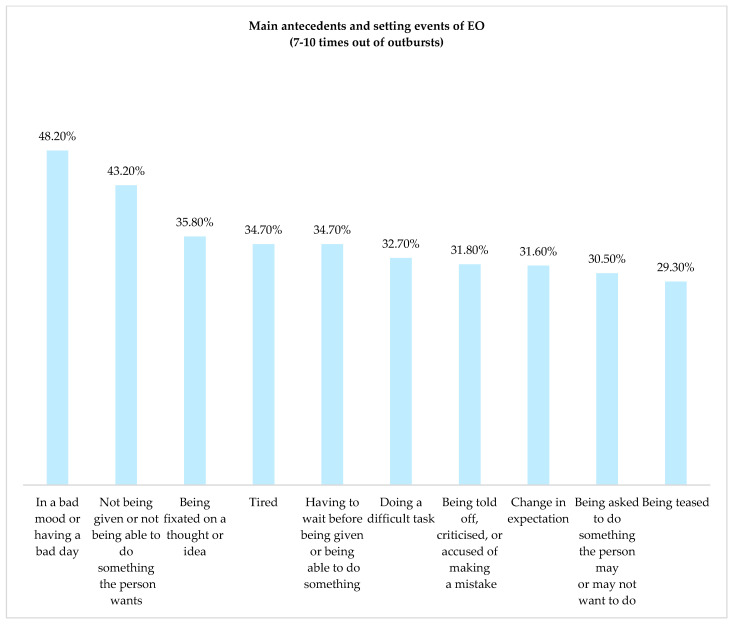
Distribution of the main clinical, emotional, and contextual triggers with higher frequency for the emotional outbursts in the sample (n = 689).

According to parents, the most frequent EO presented by children with ASD is the ‘behavioral indicators of emotion’ (52.0%) followed by ‘increased motor activity’ (28.3%), ‘non-speech vocalizations’, (27.6%), ‘mild verbal aggression’ (23.8%), and ‘avoidance’ (21.5%) (Figure 1). When analyzing antecedents and setting events of outbursts (clinical, emotional, and contextual factors) that provoke EOs in our sample, it is noticeable that 48.2% of children frequently or always exhibit ‘bad mood’ as a trigger or an antecedent for EOs. Additionally, the following motivations for EOs were frequently or always reported by at least one-quarter of the caregivers: ‘Not being given or not being able to do something the person wants’; ‘Being fixated on a thought or idea’; ‘Being tired’; ‘Having to wait before being given/being able to do something’; ‘Doing a difficult task’; ‘Being told off, criticized, or accused of making a mistake’; ‘Changes in his/her expectation’; ‘Being asked to do something he/she may not want to do’; ‘Being teased’ (Figure 2).

For the next step, we selected the most common EOs that had been identified in the children with ASD in our sample, specifically those with a percentage of occurrence greater than or equal to 15%, scored as frequently or always. Aiming to identify predictors of these more common EOs, we listed the following variables that were classified as the most frequent, with a percentage greater than or equal to 25% in the frequently/always category: the level of verbal ability, disability, age at diagnosis, age at the start of treatment, weekly hours of treatment, and emotional, clinical, and contextual factors.

Among the various factors tested (logit models the emotional factor), the emotional factor ‘in a bad mood or having a bad day’ was the only one that significantly increased the likelihood of all types of EO occurring in children with ASD in our sample (Table 2). Therefore, if a child was sometimes in a bad mood or having a bad day there was an increased probability for displaying (in descending order): mild verbal aggression/i.e., insults, name-calling, screaming, shouting, and swearing (odd ratio = 4.84, *p* < 0.001); talking to self and others/i.e., agitated, repetitive speech, and self-deprecating speech (odd ratio = 4.60, *p* < 0.001); avoidance behavior/i.e., dropping to the floor, going to another room, leaving the situation, running away (odd ratio = 3.95, *p* < 0.001); behavioral indicators of emotion/i.e., angry or annoyed facial expressions, crying, signs of distress, and whining (odd ratio = 3.48, *p* < 0.001 for frequently/always); non-speech vocalizations/i.e., making sounds or noises (odd ratio = 3.40, *p* < 0.001); mild aggression towards property/i.e., breaking objects, smashing windows, and throwing objects dangerously (odd ratio = 2.90, *p* = 0.001); and increased physiological arousal/i.e., red face, salivating, sweating (odd ratio = 2.30, *p* = 0.005).

In addition, other factors were relevant for specific groups of children. For children who do not speak or use only single words, there is also a significant increased probability for several types of EO. For example: ‘non-speech vocalizations’; odd ratio = 4.63 (*p* < 0.001) when the child does not speak; odd ratio = 4.61 (*p* < 0.001) when the child uses only single words to speak; ‘increased physiological arousal’ (odd ratio = 2.57, *p* = 0.043, when the child uses only single words to speak; odd ratio = 2.21, *p* = 0.043, when the child does not speak), and ‘avoidance behavior’ (odd ratio = 2.39, *p* = 0.004, when the child uses only single words to speak). The EO ‘non-speech vocalizations’ also increases the chances of occurrence in children who receive between 11 and 15 h (odd ratio = 2.58, *p* = 0.023) and between 16 and 20 h of treatment (odd ratio = 2.57, *p* = 0.023). The personal factor ‘being fixated on a thought or idea’ also increased the chances of ‘talking to self and others’ (odd ratio = 3.30, *p* = 0.001 for frequently/always), and showing ‘mild verbal aggression’ (odd ratio = 2.37, *p* = 0.009 for sometimes and odd ratio = 2.01, *p* < 0.009 for frequently/always). The factor ‘having to wait before being given or being able to do something’, frequently/always increases the odds of occurrence of ‘mild aggression towards property’ (odd ratio = 2.18, *p* = 0.033) and ‘non-speech vocalizations’ (odd ratio = 2.00, *p* = 0.042). Being told off, criticized, or accused of making a mistake increases the odds of occurrence of increased ‘physiological arousal’ (odd ratio = 2.38, *p* = 0.008) and ‘behavioral indicators of emotion’ (odd ratio = 2.10, *p* = 0.043). Change in expectation increases the probability of occurrence of ‘behavioral indicators of emotion’ (odd ratio = 2.15, *p* = 0.043). The factor being asked to do something the person may or may not want to do also increases the probability of occurrence of three types of ‘mild aggressive behaviors’: ‘mild verbal aggression’ (odd ratio = 2.65, *p* = 0.005), ‘mild aggression towards property’ (odd ratio = 2.39, *p* = 0.018), and ‘mild physical aggression towards others without physical injury’ (odd ratio = 2.27, *p* = 0.027). Being teased increases the chances of ‘mild verbal aggression’ occurring (odd ratio = 2.58, *p* = 0.003 for frequently/always); mild aggression towards property (odd ratio = 2.43, *p* = 0.002 for frequently/always and odd ratio = 2.09, *p* = 0.002 for sometimes); and ‘mild physical aggression towards others without physical injury’ (odd ratio = 2.90, *p* = 0.001 for frequently/always and odd ratio = 2.09, *p* = 0.001 for sometimes). The factor ‘change in own routine’ increases the probability of occurrence of the ‘increased motor activity’ (odd ratio = 2.38, *p* = 0.006 (Table 2).

## 4. Discussion

This study aimed to characterize the profile of EOs in children with ASD across five countries in Latin America and to identify clinical, emotional, and contextual factors that contribute to the onset and frequency. We found that, among the five countries, the EO ‘behavioral indicators of emotion’ was most reported by the caregivers with more than 50% of children experiencing such EOs. We found a large heterogeneity of EOs in the sample characterized mainly by topographies such us angry; annoyed facial expressions; crying; signs of distress; aggression toward people, property, or self; and whining, among others.

According to parents, the children in this study presented significant variability in contextual and individual factors, with more than 50 factors identified, 34 of which were always or almost always triggers of EOs (see Figure 2). The most frequent triggers were bad mood or having a bad day, not being given or not being able to do something the person wants, being fixated on a thought or idea, having to wait before being given or being able to do something, being tired, and doing a difficult task. These antecedents and setting events can be classified into a group defined as ‘States’ (setting events related to the physiological state of the individual, e.g., tired) or ‘Cognitive Demand’ (antecedents that may be cognitively demanding for an individual, e.g., change in expectation) [10]. In our sample, we identified triggers from the two clusters described by Chung et al. [10], with frequencies of occurrence that were higher than or equal to 25% in the frequently/always category.

Evidence has shown that children with ASD have difficulties with emotional and behavioral regulation, generally mediated by cognitive factors [46,47], physiological factors [48], sensory factors [49], and inadequate behavioral management [50]. This clinical profile makes them vulnerable when exposed to negative environmental settings that become triggers for EOs [51,52,53]. Assessment tools such as the EOQ allow researchers to evaluate specific characteristics and topographies of EOs, as well as clinical, emotional, and contextual antecedents [10]. Our results showed how common these clinical, emotional, and contextual factors are in participants among the five Latin American countries, highlighting how families are able to identify a wide variability of contextual and individual factors that are similar among children from different countries. The identification of these factors can help in planning psychosocial interventions aimed at caregivers that help the emotional and behavioral management of children with ASD and improve emotional dysregulation and irritability in ASD. This would make it possible to design a multimodal treatment plan as recommended by Salazar et al. [3].

Thus, the wide variability of contextual and individual factors identified in this study underscores the need for parents to be guided in making them aware of the negative influence of these environmental and emotional factors. In autism, the importance of parental training has been proven in several studies [54]. For example, O’Nions et al. [51] carried out a meta-synthesis that explored qualitative data on parenting practices in response to externalizing behaviors in children with ASD. The authors found a range of parenting strategies used by parents to support their child’s behaviors, including accommodating the child, modifying the environment, providing structure and routine, supervision and monitoring, managing non-compliance with everyday activities, responding to externalizing behaviors, managing distress, maintaining safety, and analyzing and planning. This study found that accommodating the child’s needs was the most common strategy used by parents in response to externalizing behaviors. However, this review was limited to qualitative analysis of case studies and qualitative studies, all with samples from developed countries [51]. Therefore, our study provides a novel contribution to the region by highlighting the need for interventions aimed at parenting strategies to manage common problem behaviors in ASD in Latin American countries. These strategies may include the provision of routine, structure, contingent reinforcement, management of distraction/stimulation, and reduction of uncertainty [55].

It was noticeable that being in a bad mood or having a bad day was a single factor that increased the chances of occurrence of all types of EO. This can be interpreted as an indicator of irritability. Although the conceptualization of irritability is still controversial [56] and the underlying mechanisms are not well-known [57], it is well-established that it is usually associated with the emission of outbursts, especially in children with ASD [58]. In our study, the probability of occurrence of different EOs showed an association with ‘bad mood or having a bad day’ (e.g., mild verbal aggression, talking to self and others, avoidance behavior, behavioral indicators of emotion, non-speech vocalizations, mild aggression towards property, and increased physiological arousal) when the child is in a bad mood or having a bad day ranged between 2.30 and 4.84 (see Table 2)

Our findings emphasize the significant role of irritability/bad mood in the occurrence of EO among children with ASD. Monitoring and addressing these emotional factors are crucial for improving the mental health and well-being of this population. Our data also reveal how irritability should not be disregarded or underestimated when monitoring the mental health of children with ASD. Previous studies have revealed that children with ASD with other clinical conditions, such as oppositional defiant disorder, attention deficit hyperactivity disorder, and anxiety disorder, are more likely to present irritability such as a manifestation of ED. For example, a study by Carter Leno et al. [57], in a sample of 77 adolescents with ASD (mean age 15.3) reported that the affective reactivity index was significantly correlated with the irritability (r = 0.78, *p* < 0.001) and the number of oppositional defiant symptoms (r = 0.71, *p* < 0.001). A study by Pan and Yeh [59], in 56 children with ASD (mean age 10.36), also verified symptom profiles of irritability associated with emotional and conduct problems like those of disruptive mood dysregulation disorder in the nonautistic population. Considering the relevance of irritability among children, adolescents, and adults with ASD, it seems worthwhile to explore strategies and programs that can minimize these issues. Other secondary factors that we identified increased the EOs, such as limitations in expressive language skills (children who do not speak or use only single words to speak). It may be that children in this sample could benefit from augmentative and alternative communication systems [60].

The current study has several limitations. First, the convenience criteria and online data collection required participants to have access to the internet and computer literacy, which is not universal in the Latin American region. This likely introduced bias, resulting in a higher proportion of parents with educational levels above the regional average. Additionally, there was an unequal distribution of sample size and parental profile among the five countries. We relied on parental reports to register outbursts in children without observational corroboration. Furthermore, there was no control for the severity of ASD or the co-occurrence of psychiatric conditions.

## 5. Conclusions

Our findings offer important insights into EOs in Latin American children with ASD. Despite the variability in contextual and individual factors associated with EOs, our data underscore the importance of addressing irritability in children with ASD. This emotional factor was an important and common antecedent of EOs, highlighting that mental health professionals should not disregard or underestimate this trigger strongly associated with ED. The identified variability of contextual and individual factors that preceded EOs in the sample supports the theoretical model of clusters of factors, suggesting the need for multimodal interventions involving parental training to identify, manage, and monitor these factors.

Future cross-cultural studies should explore our findings in other contexts, particularly in schools, to contribute to the development of regional strategies and tools for understanding and preventing EO in different environments. These studies should investigate the role of EOs in school settings and support collaborative actions between families, teachers, and the educational community.

## Figures and Tables

**Table 1:** Sociodemographic and clinical characteristics of caregivers and children with autism spectrum disorder by country (n = 689).

	Country					Total	*p*
	Argentina	Uruguay	Brazil	Chile	Dominican Republic		
Characteristics of the caregivers							
Age (years)							**0.003 ^a^**
Average ± SD	42.0 ± 6.6 ^ⱡ^	42.6 ± 9.1 ^ⱡ^	43.2 ± 5.6 ^ⱡ^	42.2 ± 6.9 ^ⱡ^	38.3 ± 7.9 ^ƪ^	42.2 ± 7.1	
Median (Minimum and Maximum)	42.0 (37.3 to 45.0)	42.0 (36.0 to 49.0)	43.0 (39.0 to 47.0)	43.0 (38.0 to 47.0)	39.0 (32.5 to 44.0)	42.0 (38.0 to 46.0)	
n	244	126	151	101	40	662	
Educational level of the caregiver							**<0.001 ^d^**
Nonliterate	4/254 (1.6)	1/127 (0.8)	0/151 (0.0)	0/104 (0.0)	3/49 (6.1)	8/685 (1.2)	
Elementary School—Primary (incomplete)	4/254 (1.6)	0/127 (0.0)	1/151 (0.7)	1/104 (1.0)	6/49 (12.2)	12/685 (1.8)	
Elementary School—Primary (complete)	2/254 (0.8)	19/127 (15.0)	3/151 (2.0)	0/104 (0.0)	2/49 (4.1)	26/685 (3.8)	
Elementary School—Secondary	41/254 (16.1)	60/127 (47.2)	1/151 (0.7)	1/104 (1.0)	13/49 (26.5)	116/685 (16.9)	
High school	34/254 (13.4)	15/127 (11.8)	19/151 (12.6)	12/104 (11.5)	7/49 (14.3)	87/685 (12.7)	
Higher Education	108/254 (42.5)	23/127 (18.1)	40/151 (26.5)	62/104 (59.6)	12/49 (24.5)	245/685 (35.8)	
Post-graduate	61/254 (24.0)	9/127 (7.1)	87/151 (57.6)	28/104 (26.9)	6/49 (12.2)	191/685 (27.9)	
Relation to child							**0.006 ^d^**
Mother	225/253 (88.9)	104/127 (81.9)	138/150 (92.0)	91/105 (86.7)	45/50 (90.0)	603/685 (88.0)	
Father	9/253 (3.6)	14/127 (11.0)	10/150 (6.7)	9/105 (8.6)	2/50 (4.0)	44/685 (6.4)	
Grandmother/Grandfather	3/253 (1.2)	6/127 (4.7)	0/150 (0.0)	1/105 (1.0)	0/50 (0.0)	10/685 (1.5)	
Other	16/253 (6.3)	3/127 (2.4)	2/150 (1.3)	4/105 (3.8)	3/50 (6.0)	28/685 (4.1)	
Characteristics of the child							
Sex							0.055 ^d^
Male	201/255 (78.8)	91/127 (71.7)	123/151 (81.5)	85/105 (81.0)	37/51 (72.5)	537/689 (77.9)	
Female	54/255 (21.2)	35/127 (27.6)	27/151 (17.9)	20/105 (19.0)	12/51 (23.5)	148/689 (21.5)	
Other	0/255 (0.0)	0/127 (0.0)	1/151 (0.7)	0/105 (0.0)	0/51 (0.0)	1/689 (0.1)	
I prefer not to say	0/255 (0.0)	1/127 (0.8)	0/151 (0.0)	0/105 (0.0)	2/51 (3.9)	3/689 (0.4)	
Age (years)							**<0.001 ^b^**
Average ± SD	8.0 ± 2.0 ^¥^	8.9 ± 2.7 ^§^	9.0 ± 2.9 ^§^	9.4 ± 2.7 ^§^	9.4 ± 3.5 ^§^	8.7 ± 2.6	
Median (Minimum and Maximum)	8.0 (6.0 to 9.0)	8.0 (6.0 to 11.0)	8.0 (7.0 to 11.0)	9.0 (7.0 to 11.0)	9.0 (6.0 to 10.0)	8.0 (7.0 to 10.0)	
n	251	123	151	105	45	675	
Child diagnosis							**<0.001 ^d^**
Autism	27/253 (10.7)	19/127 (15.0)	51/150 (34.0)	3/105 (2.9)	10/51 (19.6)	110/686 (16.0)	
Autism Spectrum Disorder (ASD)	133/253 (52.6)	87/127 (68.5)	92/150 (61.3)	92/105 (87.6)	37/51 (72.5)	441/686 (64.3)	
Asperger syndrome	37/253 (14.6)	14/127 (11.0)	4/150 (2.7)	7/105 (6.7)	3/51 (5.9)	65/686 (9.5)	
Pervasive/pervasive developmental disorder not otherwise specified	50/253 (19.8)	4/127 (3.1)	2/150 (1.3)	3/105 (2.9)	1/51 (2.0)	60/686 (8.7)	
Other	/253 (2.4)	3/127 (2.4)	1/150 (0.7)	0/105 (0.0)	0/51 (0.0)	10/686 (1.5)	
Age at ASD diagnosis (years)							**<0.001 ^b^**
Average ± SD	3.1 ± 2.8	3.7 ± 2.3	3.3 ± 2.8	5.3 ± 4.1	3.8 ± 2.8	3.7 ± 3.0	
Median (Minimum and Maximum)	2.0 (1.0 to 4.0)	3.0 (2.0 to 5.0)	2.0 (2.0 to 4.0)	4.0 (2.0 to 8.5)	3.0 (2.0 to 5.0)	3.0 (2.0 to 5.0)	
n	251	126	151	105	49	682	
Neurological or genetic disease diagnosed							
Epilepsy							0.053 ^c^
No	209/222 (94.1)	100/112 (89.3)	135/150 (90.0)	72/78 (92.3)	32/40 (80.0)	548/602 (91.0)	
Yes	13/222 (5.9)	12/112 (10.7)	15/150 (10.0)	6/78 (7.7)	8/40 (20.0)	54/602 (9.0)	
X Fragile							0.708 ^d^
No	211/212 (99.5)	106/106 (100.0)	148/150 (98.7)	72/72 (100.0)	32/32 (100.0)	569/572 (99.5)	
Sclerosis							0.207 ^d^
No	211/213 (99.1)	105/105 (100.0)	150/150 (100.0)	73/73 (100.0)	32/33 (97.0)	571/574 (99.5)	
Yes	2/213 (0.9)	0/105 (0.0)	0/150 (0.0)	0/73 (0.0)	1/33 (3.0)	3/574 (0.5)	
Other							**0.002 ^c^**
No	188/220 (85.5)	96/109 (88.1)	112/150 (74.7)	68/91 (74.7)	32/34 (94.1)	496/604 (82.1)	
Yes	32/220 (14.5)	13/109 (11.9)	38/150 (25.3)	23/91 (25.3)	2/34 (5.9)	108/604 (17.9)	
Verbal skill level							**<0.001 ^c^**
Does not speak	30/255 (11.8)	12/126 (9.5)	16/151 (10.6)	5/105 (4.8)	13/50 (26.0)	76/687 (11.1)	
Uses only single words to speak	23/255 (9.0)	26/126 (20.6)	11/151 (7.3)	7/105 (6.7)	11/50 (22.0)	78/687 (11.4)	
Uses two- or three-word sentences	32/255 (12.5)	22/126 (17.5)	14/151 (9.3)	10/105 (9.5)	4/50 (8.0)	82/687 (11.9)	
Uses sentences of four or more words	38/255 (14.9)	17/126 (13.5)	31/151 (20.5)	15/105 (14.3)	10/50 (20.0)	111/687 (16.2)	
Uses complex sentences	132/255 (51.8)	49/126 (38.9)	79/151 (52.3)	68/105 (64.8)	12/50 (24.0)	340/687 (49.5)	
Intellectual disability							**0.009 ^c^**
No	138/255 (54.1)	74/127 (58.3)	97/151 (64.2)	65/105 (61.9)	17/49 (34.7)	391/687 (56.9)	
Yes	63/255 (24.7)	30/127 (23.6)	37/151 (24.5)	23/105 (21.9)	22/49 (44.9)	175/687 (25.5)	
Do not know	54/255 (21.2)	23/127 (18.1)	17/151 (11.3)	17/105 (16.2)	10/49 (20.4)	121/687 (17.6)	
Age at start of treatment ^1^ (years)							**<0.001 ^b^**
Average ± SD	4.0 ± 2.7	4.3 ± 2.2	3.6 ± 2.8	5.4 ± 3.9	4.4 ± 2.9	4.2 ± 2.9	
Median (Minimum and Maximum)	3.0 (2.0 to 4.0)	4.0 (3.0 to 5.0)	3.0 (2.0 to 4.0)	4.0 (2.0 to 8.0)	4.0 (3.0 to 5.0)	3.0 (2.0 to 5.0)	
n	252	126	151	105	47	681	
No treatment	3/255 (1.2)	1/127 (0.8)	0/151 (0.0)	0/105 (0.0)	2/49 (4.1)	6/687 (0.9)	
Weekly treatment hours ^1^							**<0.001 ^d^**
No treatment	17/255 (6.7)	16/127 (12.6)	3/151 (2.0)	9/105 (8.6)	15/50 (30.0)	60/688 (8.7)	
1–5 h	175/255 (68.6)	92/127 (72.4)	49/151 (32.5)	81/105 (77.1)	27/50 (54.0)	424/688 (61.6)	
6–10 h	50/255 (19.6)	17/127 (13.4)	31/151 (20.5)	7/105 (6.7)	5/50 (10.0)	110/688 (16.0)	
11–15 h	3/255 (1.2)	0/127 (0.0)	22/151 (14.6)	2/105 (1.9)	0/50 (0.0)	27/688 (3.9)	
16–20 h	3/255 (1.2)	1/127 (0.8)	19/151 (12.6)	0/105 (0.0)	0/50 (0.0)	23/688 (3.3)	
20 h or more	7/255 (2.7)	1/127 (0.8)	27/151 (17.9)	6/105 (5.7)	3/50 (6.0)	44/688 (6.4)	

*p* = descriptive level of ANOVA test (^a^), Kruskal–Wallis test (^b^), Chi-Square test (^c^), and Fisher exact test (^d^). ^1^ = behavioral intervention or applied behavior analysis, sensory integration therapy, cognitive therapy, occupational therapy, social skills training, speech therapy, medication, psychoanalysis. ^ⱡ^ and ^ƪ^ show different means according to Duncan’s multiple comparisons; ^¥^ and ^§^ show different means according to Dunn-Bonferroni multiple comparisons.

**Table 2:** Logit models for emotional outbursts ordered with random effects for all the emotional outbursts with a frequency equal to or greater than 15% in the sample (n = 689).

Emotional Outburst	Behavioral Indicators of Emotion		Increased Motor Activity		Non-Speech Vocalizations		Mild Verbal Aggression		Avoidance		Mild Aggression towards Property		Talking to Self and Others		Increased Physiological Arousal		Mild Physical Aggression towards Others	
Clinical, Emotional and Contextual Factors	RC Adjusted (95%) CI	*p*	RC Adjusted (95%) CI	*p*	RC Adjusted (95%) CI	*p*	RC Adjusted (95%) CI	*p*	RC Adjusted (95%) CI	*p*	RC Adjusted (95%) CI	*p*	RC Adjusted (95%) CI	*p*	RC Adjusted (95%) CI	*p*	RC Adjusted (95%) CI	*p*
Verbal skill level		0.158		0.169		**<** **0** **.001**		**<** **0** **.001**		**0** **.004**		0.083		**<** **0** **.001**		**0** **.043**		**0** **.007**
Does not speak	1.29 (0.62, 2.67)	0.493	1.77 (0.91, 3.46)	0.094	4.63 (2.34, 9.15)	<0.001	0.11 (0.05, 0.26)	<0.001	1.83 (0.94, 3.57)	0.074	0.88 (0.45, 1.74)	0.723	0.03 (0.01, 0.09)	<0.001	2.21 (1.13, 4.30)	0.020	2.74 (1.41, 5.33)	0.003
Uses only single words to speak	0.96 (0.48, 1.90)	0.899	1.52 (0.81, 2.86)	0.189	4.61 (2.38, 8.92)	<0.001	0.28 (0.13, 0.59)	0.001	2.39 (1.27, 4.49)	0.007	0.74 (0.37, 1.46)	0.383	0.18 (0.08, 0.40)	<0.001	2.57 (1.33, 4.94)	0.005	2.29 (1.17, 4.47)	0.015
Uses two- or three-word sentences	0.57 (0.31, 1.05)	0.070	0.97 (0.56, 1.69)	0.925	1.67 (0.95, 2.93)	0.077	0.33 (0.18, 0.60)	<0.001	1.32 (0.76, 2.31)	0.324	0.87 (0.49, 1.54)	0.622	0.19 (0.10, 0.37)	<0.001	1.55 (0.87, 2.76)	0.136	1.96 (1.10, 3.49)	0.023
Uses sentences of four or more words	0.70 (0.41, 1.19)	0.190	0.81 (0.50, 1.32)	0.407	1.25 (0.75, 2.05)	0.390	0.47 (0.28, 0.80)	0.006	0.70 (0.43, 1.15)	0.159	0.48 (0.29, 0.81)	0.006	0.50 (0.30, 0.84)	0.008	1.26 (0.76, 2.11)	0.369	1.06 (0.63, 1.78)	0.833
Intellectual disability (ref. No)		0.225		0.134		**0** **.011**		0.516		0.769		**0** **.018**		0.192		**0** **.143**		**0** **.078**
Yes	1.38 (0.85, 2.24)	0.196	1.54 (0.99, 2.38)	0.054	1.96 (1.26, 3.06)	0.003	1.31 (0.81, 2.12)	0.278	0.85 (0.55, 1.32)	0.469	1.89 (1.20, 2.97)	0.006	1.21 (0.75, 1.97)	0.431	1.55 (1.00, 2.42)	0.051	1.69 (1.07, 2.68)	0.026
I don’t know	0.87 (0.53, 1.45)	0.600	1.05 (0.66, 1.67)	0.824	1.45 (0.91, 2.32)	0.118	1.21 (0.73, 1.99)	0.458	0.93 (0.59, 1.47)	0.751	1.50 (0.94, 2.41)	0.090	0.69 (0.41, 1.18)	0.175	1.14 (0.71, 1.83)	0.593	1.36 (0.84, 2.18)	0.210
Age of ASD diagnosis	0.99 (0.88, 1.11)	0.858	0.84 (0.74, 0.95)	0.006	0.94 (0.83, 1.06)	0.299	0.99 (0.87, 1.12)	0.854	1.01 (0.90, 1.13)	0.876	0.94 (0.83, 1.07)	0.365	0.99 (0.87, 1.14)	0.935	1.00 (0.89, 1.13)	0.974	0.91 (0.80, 1.03)	0.128
Age at start of treatment (years)	0.97 (0.86, 1.09)	0.621	1.12 (0.99, 1.27)	0.072	1.06 (0.94, 1.20)	0.311	1.03 (0.91, 1.17)	0.603	1.07 (0.95, 1.21)	0.243	1.04 (0.92, 1.18)	0.537	0.99 (0.86, 1.13)	0.862	1.03 (0.91, 1.16)	0.611	1.00 (0.88, 1.14)	0.965
Weekly treatment		0.970		0.077		**0** **.023**		0.356		0.153		0.565		0.406		0.845		**0** **.034**
No treatment	1.31 (0.65, 2.65)	0.446	1.56 (0.83, 2.93)	0.171	1.44 (0.77, 2.70)	0.254	0.75 (0.38, 1.47)	0.398	1.28 (0.69, 2.36)	0.430	0.95 (0.50, 1.81)	0.870	1.59 (0.82, 3.08)	0.172	1.08 (0.57, 2.05)	0.805	1.04 (0.52, 2.09)	0.901
6, 10 h	0.91 (0.55, 1.51)	0.705	1.28 (0.81, 2.02)	0.283	1.41 (0.86, 2.31)	0.171	0.91 (0.54, 1.54)	0.736	1.35 (0.85, 2.16)	0.203	0.95 (0.58, 1.56)	0.844	1.33 (0.79, 2.23)	0.284	1.10 (0.68, 1.78)	0.698	1.51 (0.92, 2.47)	0.101
11, 15 h	0.95 (0.38, 2.35)	0.905	3.19 (1.39, 7.34)	0.006	2.58 (1.06, 6.31)	0.037	1.59 (0.64, 3.94)	0.320	2.16 (0.91, 5.17)	0.083	1.67 (0.72, 3.86)	0.229	1.37 (0.57, 3.30)	0.477	1.24 (0.51, 2.98)	0.635	0.82 (0.33, 2.06)	0.675
16, 20 h	1.14 (0.41, 3.22)	0.798	1.54 (0.66, 3.55)	0.315	2.57 (1.00, 6.61)	0.051	0.64 (0.23, 1.81)	0.402	0.48 (0.20, 1.18)	0.110	0.62 (0.25, 1.50)	0.287	2.10 (0.76, 5.78)	0.151	1.00 (0.42, 2.38)	0.996	0.71 (0.30, 1.71)	0.450
20 h or more	1.00 (0.48, 2.07)	0.996	1.05 (0.52, 2.14)	0.889	3.18 (1.52, 6.66)	0.002	1.82 (0.88, 3.74)	0.106	0.97 (0.50, 1.89)	0.936	1.42 (0.70, 2.88)	0.335	1.59 (0.70, 3.63)	0.268	1.60 (0.82, 3.14)	0.171	2.71 (1.39, 5.27)	0.003
Triggers																		
In bad mood or having a bad day		**<** **0** **.001**		**0** **.005**		**<** **0** **.001**		**<** **0** **.001**		**<** **0** **.001**		**0** **.001**		**<** **0** **.001**		**0** **.011**		**0** **.001**
Sometimes	1.53 (0.86, 2.74)	0.150	1.20 (0.66, 2.18)	0.545	1.41 (0.76, 2.61)	0.275	2.23 (1.02, 4.89)	0.045	2.10 (1.12, 3.92)	0.020	1.49 (0.75, 2.94)	0.255	2.32 (1.10, 4.89)	0.027	1.24 (0.65, 2.36)	0.508	1.03 (0.52, 2.04)	0.941
Often/Always	3.48 (1.81, 6.73)	<0.001	2.30 (1.21, 4.35)	0.011	3.40 (1.74, 6.65)	<0.001	4.84 (2.16, 10.83)	<0.001	3.95 (2.04, 7.63)	<0.001	2.90 (1.45, 5.84)	0.003	4.60 (2.11, 10.02)	<0.001	2.24 (1.14, 4.37)	0.019	2.35 (1.16, 4.79)	0.018
Not being given or not being able to do something the person		**0.048**		0.136		0.115		0.316		0.960		0.612		0.635		0.504		0.074
Sometimes	1.77 (1.01, 3.11)	0.047	1.63 (0.93, 2.86)	0.085	1.85 (1.03, 3.32)	0.038	1.30 (0.68, 2.49)	0.429	1.09 (0.61, 1.95)	0.776	1.25 (0.68, 2.27)	0.473	0.99 (0.53, 1.87)	0.980	0.89 (0.50, 1.60)	0.700	2.08 (1.10, 3.92)	0.024
Often/Always	2.21 (1.16, 4.21)	0.016	1.18 (0.64, 2.18)	0.605	1.57 (0.83, 2.97)	0.165	1.66 (0.84, 3.31)	0.148	1.07 (0.57, 1.99)	0.842	1.38 (0.73, 2.59)	0.322	0.79 (0.40, 1.57)	0.501	0.72 (0.38, 1.34)	0.297	1.89 (0.97, 3.67)	0.060
Being fixated on a thought or idea		**0** **.021**		0.352		0.942		**0** **.009**		0.116		**0** **.031**		**0** **.001**		**0** **.002**		0.078
Sometimes	0.98 (0.61, 1.59)	0.948	1.35 (0.85, 2.17)	0.205	1.08 (0.66, 1.77)	0.748	2.37 (1.37, 4.10)	0.002	1.56 (0.97, 2.51)	0.067	1.09 (0.66, 1.80)	0.742	1.77 (1.01, 3.10)	0.046	0.85 (0.52, 1.39)	0.517	1.37 (0.82, 2.28)	0.233
Often/always	1.93 (1.09, 3.43)	0.025	1.43 (0.85, 2.40)	0.184	1.08 (0.64, 1.85)	0.765	2.01 (1.11, 3.65)	0.021	1.65 (0.99, 2.77)	0.056	1.84 (1.07, 3.17)	0.027	3.30 (1.78, 6.12)	<0.001	1.89 (1.11, 3.20)	0.019	1.87 (1.08, 3.24)	0.025
Tired		0.72		0.639		0.221		0.435		**0** **.022**		0.412		0.756		0.124		0.915
Sometimes	1.39 (0.83, 2.32)	0.206	1.21 (0.74, 1.99)	0.445	1.57 (0.93, 2.65)	0.090	1.30 (0.73, 2.29)	0.374	1.58 (0.96, 2.62)	0.074	1.45 (0.84, 2.51)	0.184	0.82 (0.47, 1.43)	0.495	1.60 (0.94, 2.72)	0.081	1.13 (0.64, 1.99)	0.675
Often/Always	2.13 (1.11, 4.08)	0.023	1.32 (0.73, 2.38)	0.354	1.34 (0.72, 2.49)	0.363	0.99 (0.51, 1.92)	0.977	2.84 (1.56, 5.17)	0.001	1.37 (0.73, 2.56)	0.323	0.93 (0.49, 1.75)	0.813	1.85 (1.01, 3.41)	0.047	1.09 (0.57, 2.09)	0.787
Having to wait before being given or being able to do something		0.170		0.439		**0** **.042**		0.457		0.414		**0** **.033**		0.604		0.062		0.188
Sometimes	1.60 (0.97, 2.64)	0.065	1.19 (0.72, 1.94)	0.499	1.85 (1.11, 3.07)	0.018	1.39 (0.81, 2.40)	0.234	1.12 (0.68, 1.83)	0.661	1.73 (1.03, 2.91)	0.039	1.24 (0.72, 2.12)	0.440	1.63 (0.97, 2.71)	0.063	1.56 (0.91, 2.67)	0.106
Often/Always	1.57 (0.84, 2.95)	0.161	1.46 (0.81, 2.61)	0.206	2.00 (1.10, 3.64)	0.024	1.42 (0.76, 2.65)	0.267	1.44 (0.80, 2.57)	0.222	2.18 (1.21, 3.94)	0.010	1.01 (0.54, 1.88)	0.982	2.02 (1.12, 3.66)	0.020	1.72 (0.93, 3.16)	0.082
Doing a difficult task		0.056		0.921		0.102		0.400		0.102		0.061		0.421		0.917		0.625
Sometimes	0.68 (0.41, 1.11)	0.121	1.05 (0.66, 1.68)	0.839	0.67 (0.42, 1.08)	0.102	1.20 (0.71, 2.03)	0.496	1.41 (0.89, 2.22)	0.144	0.63 (0.39, 1.02)	0.058	0.71 (0.42, 1.22)	0.217	1.00 (0.62, 1.62)	0.994	0.84 (0.51, 1.39)	0.506
Often/Always	0.48 (0.27, 0.88)	0.016	0.96 (0.57, 1.64)	0.890	0.56 (0.33, 0.96)	0.036	0.89 (0.50, 1.57)	0.681	0.93 (0.55, 1.56)	0.772	0.53 (0.31, 0.91)	0.021	0.86 (0.48, 1.54)	0.617	0.92 (0.54, 1.57)	0.753	0.76 (0.43, 1.33)	0.333
Being told off criticized or accused of making a mistake		**0** **.043**		**0** **.043**		**0** **.186**		**0** **.340**		**0** **.189**		**0** **.604**		**0** **.162**		**0** **.008**		**0** **.654**
Sometimes	1.62 (0.99, 2.66)	0.057	1.64 (1.03, 2.61)	0.038	1.49 (0.92, 2.43)	0.105	1.47 (0.87, 2.48)	0.145	1.42 (0.89, 2.24)	0.137	1.16 (0.71, 1.89)	0.548	1.68 (0.98, 2.90)	0.060	1.77 (1.08, 2.89)	0.022	0.93 (0.57, 1.54)	0.786
Often/Always	2.10 (1.14, 3.87)	0.017	1.91 (1.11, 3.28)	0.020	1.07 (0.61, 1.89)	0.809	1.36 (0.76, 2.43)	0.305	1.59 (0.93, 2.72)	0.089	1.33 (0.76, 2.32)	0.316	1.57 (0.84, 2.92)	0.154	2.38 (1.37, 4.14)	0.002	0.77 (0.44, 1.38)	0.383
Change in expectation		0.056		0.841		**0** **.039**		0.072		0.949		0.901		0.489		0.335		0.495
Sometimes	1.16 (0.70, 1.92)	0.557	0.97 (0.59, 1.58)	0.887	0.54 (0.32, 0.91)	0.021	0.63 (0.36, 1.09)	0.096	1.07 (0.65, 1.77)	0.783	0.90 (0.54, 1.51)	0.692	0.85 (0.48, 1.52)	0.591	1.42 (0.83, 2.42)	0.204	0.75 (0.44, 1.28)	0.289
Often/Always	2.15 (1.09, 4.23)	0.027	1.11 (0.60, 2.03)	0.742	0.77 (0.41, 1.46)	0.431	1.01 (0.52, 1.94)	0.981	1.10 (0.60, 2.02)	0.753	0.87 (0.47, 1.62)	0.662	1.14 (0.58, 2.25)	0.710	1.12 (0.60, 2.09)	0.725	0.89 (0.47, 1.68)	0.725
Being asked to do something the person may or may not want to do		0.842		0.062		0.069		**0.005**		0.470		**0.018**		0.504		0.192		**0.027**
Sometimes	1.08 (0.64, 1.83)	0.761	1.16 (0.72, 1.89)	0.538	0.88 (0.53, 1.47)	0.621	1.35 (0.77, 2.36)	0.297	1.19 (0.73, 1.95)	0.479	1.39 (0.82, 2.35)	0.221	0.91 (0.53, 1.58)	0.751	0.79 (0.48, 1.31)	0.366	1.36 (0.79, 2.32)	0.262
Often/Always	1.22 (0.62, 2.39)	0.561	1.89 (1.04, 3.41)	0.036	1.51 (0.81, 2.81)	0.197	2.65 (1.36, 5.17)	0.004	1.45 (0.80, 2.62)	0.224	2.39 (1.26, 4.53)	0.007	1.22 (0.62, 2.38)	0.561	1.18 (0.64, 2.19)	0.590	2.27 (1.20, 4.30)	0.012
Being teased		0.512		0.486		0.223		**0.003**		0.459		**0.002**		0.840		0.103		**0.001**
Sometimes	0.75 (0.46, 1.23)	0.253	1.29 (0.83, 2.02)	0.262	0.98 (0.62, 1.57)	0.947	1.36 (0.83, 2.24)	0.219	1.32 (0.83, 2.07)	0.238	2.09 (1.31, 3.34)	0.002	0.91 (0.54, 1.52)	0.710	1.64 (1.03, 2.63)	0.037	2.09 (1.30, 3.38)	0.003
Often/Always	0.79 (0.44, 1.43)	0.436	1.08 (0.64, 1.80)	0.782	1.46 (0.86, 2.48)	0.165	2.58 (1.48, 4.52)	0.001	1.10 (0.65, 1.86)	0.714	2.43 (1.42, 4.16)	0.001	1.04 (0.59, 1.84)	0.894	1.24 (0.74, 2.09)	0.412	2.90 (1.68, 5.01)	<0.001
Hungry or thirsty		0.759		0.211		0.543		0.624		0.629		0.658		0.723		**0.002**		0.875
Sometimes	1.19 (0.75, 1.88)	0.459	0.84 (0.55, 1.29)	0.431	1.10 (0.71, 1.71)	0.659	1.18 (0.74, 1.88)	0.484	1.03 (0.68, 1.58)	0.877	1.20 (0.77, 1.87)	0.430	0.83 (0.52, 1.32)	0.427	0.48 (0.31, 0.76)	0.002	1.01 (0.64, 1.60)	0.972
Often/Always	1.11 (0.60, 2.06)	0.738	1.27 (0.75, 2.16)	0.374	1.35 (0.78, 2.34)	0.278	1.31 (0.75, 2.30)	0.342	0.83 (0.49, 1.40)	0.483	1.26 (0.73, 2.15)	0.404	0.85 (0.49, 1.50)	0.577	0.42 (0.25, 0.72)	0.002	1.13 (0.65, 1.97)	0.659
Disagreement with others		0.660		0.174		0.361		0.363		0.589		0.164		0.102		0.906		0.630
Sometimes	1.15 (0.71, 1.88)	0.563	0.98 (0.63, 1.54)	0.944	1.40 (0.88, 2.23)	0.156	0.99 (0.60, 1.62)	0.969	0.95 (0.61, 1.47)	0.805	1.04 (0.65, 1.66)	0.861	1.57 (0.94, 2.61)	0.085	1.10 (0.69, 1.77)	0.682	1.27 (0.78, 2.06)	0.337
Often/Always	0.91 (0.48, 1.72)	0.773	0.64 (0.37, 1.11)	0.115	1.33 (0.75, 2.35)	0.333	0.71 (0.39, 1.27)	0.250	0.76 (0.44, 1.32)	0.333	0.66 (0.37, 1.16)	0.150	1.01 (0.56, 1.83)	0.968	1.11 (0.64, 1.95)	0.705	1.19 (0.68, 2.11)	0.543
Change in own routine		0.466		**0.006**		0.321		0.367		0.674		0.486		0.141		0.158		0.507
Sometimes	1.26 (0.81, 1.95)	0.306	1.66 (1.09, 2.52)	0.017	1.04 (0.67, 1.61)	0.850	1.17 (0.74, 1.87)	0.497	1.09 (0.71, 1.67)	0.705	1.29 (0.83, 2.00)	0.267	1.59 (1.00, 2.55)	0.052	1.25 (0.81, 1.94)	0.314	1.31 (0.83, 2.08)	0.248
Often/Always	0.99 (0.54, 1.81)	0.974	2.38 (1.39, 4.06)	0.002	0.74 (0.43, 1.29)	0.289	0.85 (0.48, 1.53)	0.598	0.90 (0.52, 1.55)	0.701	1.10 (0.64, 1.91)	0.726	1.31 (0.73, 2.36)	0.370	1.69 (0.98, 2.91)	0.057	1.20 (0.68, 2.11)	0.525
Female (ref. Male)	1.07 (0.69, 1.67)	0.763	1.09 (0.72, 1.63)	0.692	1.00 (0.66, 1.52)	0.992	1.37 (0.88, 2.13)	0.158	0.73 (0.49, 1.09)	0.126	0.89 (0.59, 1.35)	0.582	0.81 (0.51, 1.28)	0.363	1.21 (0.79, 1.85)	0.379	1.21 (0.79, 1.87)	0.383
Child’s age (years)	0.98 (0.91, 1.05)	0.564	1.03 (0.96, 1.11)	0.356	0.99 (0.92, 1.06)	0.787	0.96 (0.89, 1.04)	0.283	0.94 (0.88, 1.01)	0.102	0.97 (0.91, 1.04)	0.443	1.00 (0.93, 1.08)	0.967	1.02 (0.96, 1.10)	0.487	0.96 (0.90, 1.04)	0.315
Educational of the person responsible		0.553		0.691		0.668		0.018		0.930		0.149		0.284		0.098		0.186
Elementary School I (incomplete)	1.15 (0.28, 4.77)	0.846	0.89 (0.25, 3.15)	0.860	0.67 (0.17, 2.67)	0.569	1.39 (0.33, 5.95)	0.653	1.64 (0.47, 5.74)	0.435	1.00 (0.25, 4.02)	0.995	0.79 (0.20, 3.15)	0.739	0.22 (0.05, 0.96)	0.043	1.05 (0.26, 4.29)	0.948
Elementary School I (complete)	1.07 (0.39, 2.97)	0.896	0.65 (0.25, 1.68)	0.375	1.23 (0.43, 3.52)	0.693	4.00 (1.45, 11.07)	0.007	1.19 (0.48, 2.92)	0.706	2.72 (1.03, 7.16)	0.043	2.31 (0.90, 5.91)	0.081	0.45 (0.16, 1.25)	0.124	1.03 (0.36, 3.00)	0.951
Elementary School II	0.83 (0.46, 1.49)	0.537	0.91 (0.56, 1.48)	0.707	1.27 (0.74, 2.17)	0.387	2.29 (1.35, 3.88)	0.002	0.92 (0.56, 1.50)	0.733	1.66 (1.00, 2.76)	0.049	1.05 (0.61, 1.82)	0.860	0.75 (0.45, 1.23)	0.254	1.34 (0.81, 2.23)	0.258
High school	0.73 (0.42, 1.27)	0.267	0.75 (0.45, 1.25)	0.268	0.89 (0.53, 1.48)	0.644	1.45 (0.84, 2.49)	0.180	1.02 (0.62, 1.67)	0.951	1.15 (0.68, 1.94)	0.593	0.84 (0.48, 1.48)	0.554	0.76 (0.45, 1.29)	0.313	0.76 (0.44, 1.31)	0.326
Post-graduate	1.37 (0.85, 2.20)	0.200	1.20 (0.79, 1.80)	0.389	1.08 (0.70, 1.68)	0.719	1.07 (0.68, 1.69)	0.757	0.92 (0.61, 1.40)	0.702	1.42 (0.93, 2.17)	0.103	1.51 (0.96, 2.36)	0.074	1.19 (0.78, 1.81)	0.418	1.48 (0.95, 2.28)	0.080
Non-literate	1.16 (0.13, 10.26)	0.895	0.77 (0.10, 6.11)	0.804	6.91 (0.56, 84.51)	0.130	0.00 (-)	0.999	0.40 (0.06, 2.92)	0.369	0.23 (0.02, 2.64)	0.235	0.00 (-)	0.994	0.36 (0.04, 3.16)	0.357	0.15 (0.01, 2.57)	0.191

Note: For this analysis, the effects of the variables sex of the child (adopting the male sex as a reference), the child’s age in years, and the caregiver’s education level (adopting the higher education level as a reference) were controlled; *p* values statistically significant in bold.

## Data Availability

The datasets analyzed for this study can be found in the Harvard Dataverse https://doi.org/10.7910/DVN/VURGDI, accessed on 18 September 2024.
